# New structural determinants of charged local anaesthetic block of voltage-gated sodium channels

**DOI:** 10.1186/2050-6511-13-S1-A70

**Published:** 2012-09-17

**Authors:** Péter Lukács, René Cervenka, Vaibhavkumar S Gawali, Xaver Koenig, Ágnes K Mike, Lena Rubi, Karlheinz Hilber, Hannes Todt

**Affiliations:** 1 Department of Neurophysiology and Neuropharmacology, Center for Physiology and Pharmacology, Medical University of Vienna, 1090 Vienna, Austria

## Background

Some blockers of voltage-gated Na^+^ and Ca^2+^ channels are assumed to pass through the membrane and then bind to amino acids in the internal vestibule by access from the internal side of the membrane. However, in the heart isoform of the voltage-gated Na^+^ channel, in L-type calcium channels and in T-type calcium channels an additional external access pathway (EAP) through the protein has been suggested. Furthermore, in voltage-gated Na^+^ channels (Na_V_) mutations at a specific site in the middle of the domain IV transmembrane segment 6 (site 1575 in rNa_V_1.4, 1760 in rNa_V_1.4) open an EAP for QX-222, a permanently charged, hydrophilic lidocaine analogue. Recently, the first crystal structure of a Na_V_ was published [1]. In this bacterial channel structure (Na_V_Ab) the side chain homologous to rNa_V_1.4 I1575 (I202 in Na_V_Ab) is in close contact with a pore-loop sidechain, homologous to rNa_V_1.4 W1531 (W179 in Na_V_Ab). In contrast, in all currently available structural homology models of Na_V_, W1531 is not in contact with I1575. If W1531 were positioned as suggested in the Na_V_Ab structure then a reduction in the length of the side chain at this site would be predicted to open the EAP. To test this hypothesis we generated the mutations W1531A and W1531G and tested these constructs for block by external QX-222.

## Methods

Whole-cell patch clamp measurements were done on TsA 201 cells transiently transfected with plasmids coding the rNa_V_1.4 α subunit and its mutants, the sodium channel β1 subunit and GFP. Block levels were derived at 2 Hz stimulation frequency from a holding potential of −120 mV.

## Results

Mutations W1531A and W1531G were found to be sensitive to extracellular QX-222 (block: 20.6 ± 2% and 17.7 ± 3.5%, respectively).

## Conclusions

Our results indicate that position 1531 is an important part of the EAP in rNa_V_1.4, as predicted from the crystal structure of Na_V_Ab. Thus the bacterial channel Na_V_Ab appears to share important structural motifs with eukaryotic sodium channels.
